# Effects of *Nostoc sphaeroids* Kütz polysaccharide on renal fibrosis in high‐fat mice

**DOI:** 10.1002/fsn3.2703

**Published:** 2022-03-21

**Authors:** Litao Yang, Yinlu Liu, Cuicui Bi, Bo Zhang

**Affiliations:** ^1^ Beijing Key Laboratory of Bioactive Substances and Functional Foods Beijing Union University College of Biochemical Engineering Beijing China

**Keywords:** high‐fat diet, inflammation, lipid‐decreasing, *Nostoc sphaeroids* Kütz polysaccharide, renal fibrosis

## Abstract

In this study, we investigated the effects of *Nostoc sphaeroids* Kütz polysaccharide (NSKP) on renal fibrosis in high‐fat mice. ApoE^−/−^ male mice were randomly divided into four groups: control (Cont) group, high‐fat diet (HFD) group, HFD+0.4 g/kg BW NSKP, and HFD+0.8 g/kg BW NSKP (NSKP groups). The Cont was fed a standard diet. The HFD group was fed HFD. Every day, NSKP groups were fed HFD, as well as given 0.4 g/kg BW or 0.8 g/kg BW NSKP. After 22 weeks, the serum biochemical indices (TC, TG, LDL‐C, HDL‐C, GLU, BUN, and SCR) were measured. For the kidney, the histopathological sections were observed and analyzed, and inflammatory factors and markers of renal fibrosis were measured. For the NSKP groups, the serum TC, TG, LDL‐C, BUN, and SCR were decreased, HDL‐C significantly increased compared with the HFD group. The protein expressions of TNF‐α, IL‐1β, and TGF‐β1 were significantly downregulated. The α‐SMA in renal cortex was decreased, and the mRNA expression of Col‐I and Col‐IV in renal collagen fibers was downregulated. To sum up, NSKP reduced the blood lipid of HFD mice, downregulated the inflammation of kidney, inhibited the expression of collagen fiber, and improved the renal fibrosis caused by long‐term lipid metabolism disorder.

## Graphical abstract

*Nostoc sphaeroids* Kütz polysaccharide improved the lipid level of ApoE^−/−^ mice, reduced the renal inflammatory response of high‐fat mice, reduced the expression of renal collagen fiber, and interfered with the development of renal fibrosis. This study provided more basis for the future study of chronic kidney disease caused by high fat diet.

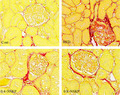

## 1 INTRODUCTION

Hyperlipidemia has become a major global health problem mainly due to excessive calorie intake and lack of exercise (Franks & McCarthy, 2016). The disorder of lipid metabolism caused by hyperlipidemia is closely related to type 2 diabetes, atherosclerosis, hypertension, nonalcoholic fatty liver, cardiovascular disease, and chronic kidney disease (Schierwagen et al., 2015; Zamani‐Garmsiri et al., 2021). With the study of “lipid nephrotoxicity,” hyperlipidemia caused by lipid disorders is a vital risk factor for the occurrence and development of chronic kidney disease (Chauhan & Vaid, 2009). Long‐term dyslipidemia can cause inflammatory activation, oxidative stress, lipid deposition, and vascular system obstruction of the kidney, and these changes will cause damage to renal structure and function (Bentley et al., 2002; Dai et al., 2014; Zhang et al., 2016).

Hyperlipidemia, especially hypercholesterolemia, plays an important role in inducing vascular and renal damage. Studies have shown that the incidence of renal disease is closely related to the increased levels of TG and TC and the abnormal lipoprotein (Reiss et al., 2015). Abnormal lipid metabolism can cause the recruitment of macrophages and promote the expression of inflammatory cytokines. Macrophage infiltration is a common feature of nephritis and renal fibrosis (Nikolic‐Paterson & Atkins, 2001). Macrophages are derived from the recruitment and local proliferation of monocytes in the blood, and always present around the myofibroblasts that present and secrete the extracellular matrix (ECM) in large numbers of α‐smooth muscle actin (α‐SMA) (Wynn & Vannella, 2016). Recent studies have found that cytokines released by macrophages cause trans‐differentiation of renal intrinsic cells (renal tubular epithelial cells and glomerular endothelial cells) and imbalance between increase and degradation of ECM, leading to renal fibrosis (Isaka, 2018; Jimenez, 2013; Lovisa et al., 2020). Studies have shown that ganoderma lucidum polysaccharides can potentially reduce renal function indices in diabetic nephropathy and alleviate renal injury (He et al., 2006). Another study found that fucoidan, a polysaccharide derived from seaweed, also had a protective effect against lipid‐induced renal injury (Fitton et al., 2015).

**FIGURE 1:**
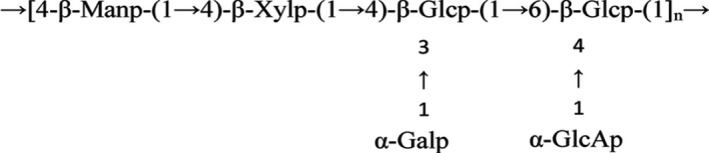
The structure of *Nostoc sphaeroids* Kütz polysaccharide (NSKP)

*Nostoc sphaeroids* Kütz is a single‐celled cyanobacterium that can form colloidal colonies composed of rosary cell filaments in a variety of environments. It belongs to the family of cyanobacteria, also known as water fungus. In Chinese fields, they grow naturally in the winter as dark green globular colonies, known in China as “*Gexianmi*,” which is used in regions such as Europe and America as a food and herbal medicine to improve people's health. *Nostoc sphaeroids* Kütz is an effective lipid‐lowering, anti‐inflammatory, and anti‐oxidation functional food (Li et al., 2019; Wei et al., 2019). *Nostoc sphaeroids* Kütz contains rich proteins, amino acids, fatty acids, vitamins, and other nutrients, among which the most important components are polysaccharide and protein. The *Nostoc sphaeroids* Kütz polysaccharides is composed of arabinose, galactose, glucose, xylose, mannose, and glucuronic acid through β‐1, 3 glycosidic bond and β‐1, 4 glycosidic bonds (Figure 1), and is an important bioactive substance (Liu et al., 2018). Our laboratory's previous research found that NSKP had the potential to treat and prevent constipation (Liu et al., 2021). Previous reports about anti‐inflammatory of NSKP also found that NSKP had antioxidant and anti‐proliferative functions (Li et al., 2018; Tang et al., 2007). There are few reports on the protective effect of NSKP on the kidney of high‐diet mice. Therefore, it is of great significance to study NSKP as a food adjuvant to intervene with diseases caused by chronic inflammation.

The purpose of this study was to explore whether NSKP can improve the chronic inflammatory response of kidney and have an interventional effect on renal fibrosis caused by high‐fat mice.

## 2 MATERIALS AND METHODS

### 2.1 Materials

*Nostoc sphaeroids* Kütz polysaccharide sample purity was 96.95% (provided by Hunan Yandi Bioengineering Co., Ltd.). Assay kits used to measure total cholesterol (TC), triglyceride (TG), high‐density lipoprotein‐cholesterol (HDL‐C), low‐density lipoprotein‐cholesterol (LDL‐C), blood urea nitrogen (BUN), serum creatinine (SCR), and glucose (GLU) in serum were purchased from Nanjing Jiancheng Bioengineering Institute. Antibodies: anti‐ACTIN(GB11230), anti‐transforming growth factor‐beta 1 (TGF‐β1, GB111876), anti‐EGF‐like module‐containing mucin‐like hormone receptor‐like 1 (EMR1 or F4/80, GB11027), anti‐interleukin‐1 beta (IL‐1β, GB11113), anti‐tumor necrosis factor‐alpha (TNF‐α, GB11188), α‐SMA (GB13044), and anti‐fibronectin (FN, GB11091), Sirius Scarlet Kit and H&E staining kit were all provided by Wuhan Servicebio Co., Ltd.

### 2.2 Animals and experiment

The Animal Research Committee of Beijing Union University approved this study (license number: 201913). Eight‐week‐old male ApoE^−/−^ mice were purchased from Beijing Charles River Experimental Animal Technology Co., Ltd. (SCXK (Jing) 2016–0006). The mice were placed in the SPF animal room of Beijing Union University and were freely provided feed and drinking water in a 12‐h light–dark cycle at a constant temperature of 22 ± 2°C.

After 40 ApoE^−/−^ mice were fed adaptively for 1 week, they were divided into four groups with 10 mice in each group. They were Cont group, HFD group, and two NSKP groups (HFD+0.4 g/kg BW NSKP and HFD+0.8 g/kg BW NSKP). Two NSKP groups were fed high‐fat diet, as well as given 0.4 g/kg BW and 0.8 g/kg BW NSKP, respectively, by gavage once a day, at 8:00 am, for 22 weeks. During the 22‐week study, the Cont group mice were fed a standard diet containing 70.18% carbohydrates, 19.47% protein, and 5% fat (Mediscience. Ltd.; Certificate No. 2018‐10030). In order to study the effect of NSKP on renal fibrosis in long‐term high‐fat mice, HFD, HFD+0.4 g/kg BW NSKP, and HFD+0.8 g/kg BW NSKP groups were fed an HFD containing 53.72% carbohydrates, 19.47% protein, and 21.46% fat (Mediscience. Ltd.; Certificate No. 2018‐10030). The dose of NSKP was determined according to the previous experimental results (Wei et al., 2019).

Bodyweight and food intake were measured during the treatment of NSKP. After 22 weeks of the experiment, all the animals were weighed. After fasting for 6 h, all the animals were injected intraperitoneally with barbital at 6 am. THE next day. Blood samples were collected. The kidneys were quickly removed, weighed, and partially fixed in 4% paraformaldehyde phosphate‐buffered solution for histopathological examination. The rest of the kidney was immediately frozen in liquid nitrogen and stored at −80°C for later experiments.

### 2.3 Measurements of serum biochemical indices

After the blood samples were collected, they were centrifuged at 4℃, 999 *g*/min, 10 min to obtain the supernatant as serum, and the kit was used to detect TC, TG, HDL‐C, LDL‐C, GLU, SRE, and BUN.

### 2.4 Histopathology

Using the kidney fixed in 4% paraformaldehyde, eight different samples were randomly selected in each group, and 4‐μm cross‐sections were prepared for hematoxylin and eosin (H&E) staining and Sirius red staining. H&E staining: sections were stained with hematoxylin and eosin, dehydrated with graded alcohol and xylene, and observed after using neutral adhesive seal. Sirius red staining: sections were stained with 0.1% Sirius red F3BA (Fisher Chemical) in saturated picric acid for 2 h, washed in 0.01 M hydrochloric acid for 2 min, and then dehydrated with graded alcohol. The morphology of renal tissue was evaluated by H&E staining and the degree of renal fibrosis was evaluated by Sirius red staining. Five fields were randomly selected using the Olympus (DP27) microscope and the percentage of positive area of fibrosis was calculated by Image Pro‐Plus 6.0 graphic analysis software.

### 2.5 Western blotting analysis

The renal cortex of the frozen kidney at −80°C was used, and eight different samples were randomly selected for each group. The cytokines TGF‐β1, F4/80, TNF‐α, and IL‐1β in renal cortex were detected. Mice renal tissues were rapidly homogenized, lysed in cold RIPA extraction buffer (Servicebio) supplemented with protease and phosphatase inhibitors, and then incubated on ice for 30 min. The protein concentration of kidney samples was measured using the BCA method (Nanjing Jiancheng Bioengineering Institute,). Protein samples were then subjected to 10% sodium dodecyl sulfate–polyacrylamide gel electrophoresis separation (SDS‐PAGE) and transferred to nitrocellulose membranes. Membranes were blocked for 1 h using 5% bovine serum albumin (BSA)/phosphate‐buffered saline (PBS), after which they were probed overnight with appropriate primary antibodies at 4℃. Blots were then washed using PBS and probed using appropriate horseradish peroxidase‐linked secondary antibodies. The membrane bands were displayed by chemiluminescence (micro‐pores), and the strip intensity of each channel was quantified by ImageJ 6.0 software.

### 2.6 Immunohistochemistry

The kidneys were fixed in 4% paraformaldehyde, and eight different samples were randomly selected for each group. The sections were prepared as described for H&E staining. Immunohistochemistry was performed using the following primary antibodies: anti‐rabbit α‐SMA, FN, TGF‐β1 antibody and then stained with goat anti‐rabbit IgG‐HRP as a secondary antibody. The reactivity of the antibodies was detected using a DAB horseradish peroxidase color development kit. The sections were counterstained with hematoxylin and observed with a microscope (Olympus DP27). Taking the appearance of brown or yellow particles as a positive expression, five non‐overlapping visual fields were randomly selected from each section, and the area optical density of the positive staining area was detected by Image Pro‐Plus 6.0 image analysis software (Area Optical Density = IOD/Area).

### 2.7 Real‐time polymerase chain reaction

The renal cortex of the frozen kidney at −80°C was used, and eight different samples were randomly selected for each group using TRIZOL (Invitrogen) to extract total RNA from mouse renal cortex. Then, we performed reverse transcription and quantitative PCR with an RT‐qPCR kit (Tiangen Biotech CO., LTD., DP304). The RT‐qPCR was carried out with DONGSHENGLONG ETC811 (Dong sheng Innovation Biotechnology Co., Ltd.). Primers for genes α‐SMA, collagen I (Col‐I), collagen IV (Col‐IV), and ACTIN were obtained from Wuhan Servicebio CO., LTD. The PCR program is as follows: 95℃ 2 min, 95℃ 20 s, 55℃ 20 s, 72℃ 20 s 30 cycles, and 72℃ 10 s. With ACTIN as the internal control, the relative expression of the genes to be tested was determined as 2^−△△CT^. Δ ΔCt = [Ct target gene (sample to be tested)‐Ct internal reference (sample to be tested)] − [Ct target gene (calibration sample)‐Ct internal reference (calibration sample)]. The sequence of primers is shown in Table 1.

**TABLE 1:** The sequences of the primers used in real‐time PCR (RT‐PCR)

Accession ID	Gene	Forward primer (5′ to 3′)	Reverse primer (3′ to 5′)
NM_007392.3	α‐SMA	TCAGGGAGTAATGGTTGGAATG	CCAGAGTCCAGCACAATACCAG
NM_001276408.1	FN	ACACGGTTTCCCATTACGCC	GGTCTTCCCATCGTCATAGCAC
NM_007742.3	Col‐I	AAGAAGCACGTCTGGTTTGGAG	GGTCCATGTAGGCTACGCTGTT
NM_009931.2	Col‐IV	CCTAACGGTTGGTCCTCACTG	GTGGGCTTCTTGAACATCTCG
NM_007393.3	β‐actin	GTGACGTTGACATCCGTAAAGA	GTAACAGTCCGCCTAGAAGCAC

### 2.8 Statistical analysis

Statistical analysis was conducted using SPSS software for windows (version 22). Data were assessed using one‐way ANOVA and Newman–Keuls pair‐wise comparison. *p*‐Values <.05 were considered significant differences. All data from these assays are shown as mean ± SEM.

## 3 RESULTS

### 3.1 Effects of NSKP on bodyweight, kidney weight, and food intake

**FIGURE 2:**
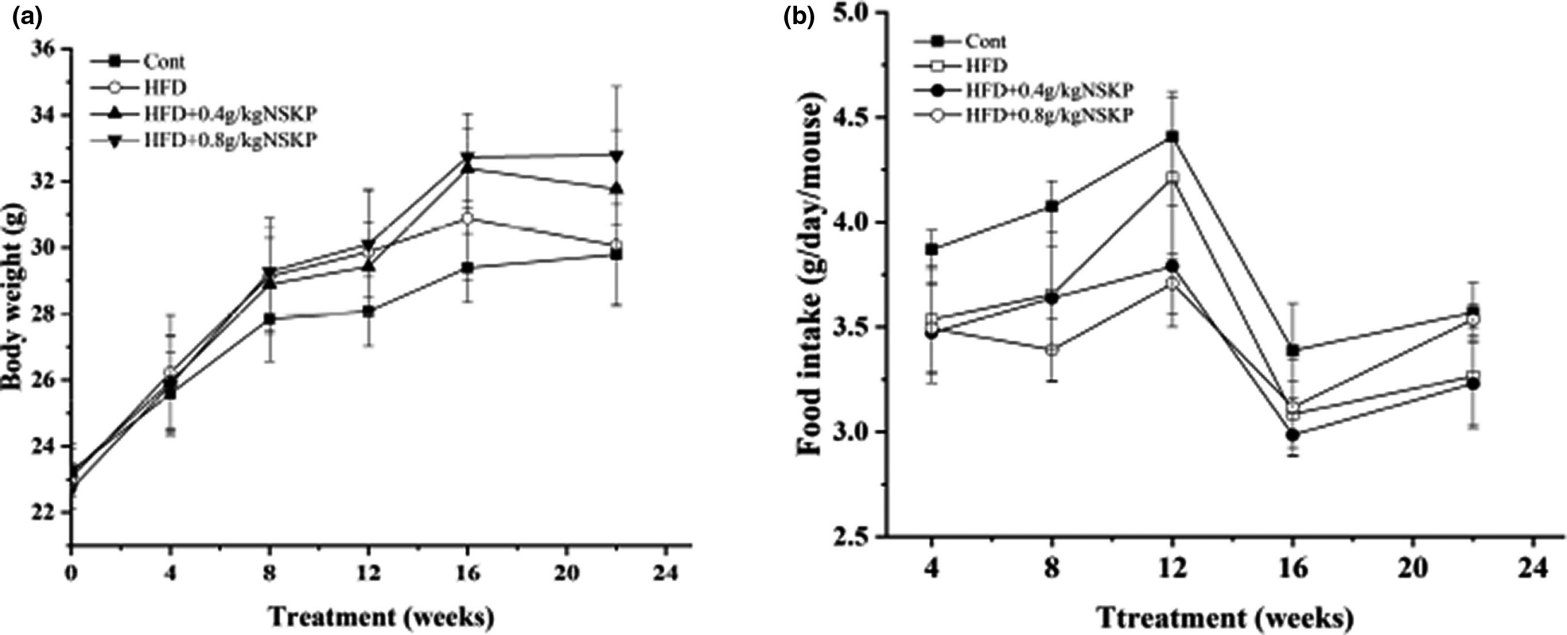
Effects of *Nostoc sphaeroids* Kütz polysaccharide (NSKP) on changes bodyweight (a) and changes food intake (b) in high‐fat mice. Values are mean ± SEM (*n* = 10)

As shown in Figure 2a,b, compared with the HFD group, the changes in bodyweight at the end of 4, 8, 12, 16, and 22 weeks of the Cont group, HFD group, and two NSKP groups had no significant difference. Meanwhile, there was no significant difference in body and kidney weight, food intake, and kidney weight/bodyweight of mice at the end of 22 weeks from NSKP groups (Table 2). The results showed that NSKP had no effect on the bodyweight, food intake, kidney weight, and kidney weight/bodyweight of high‐fat mice.

**TABLE 2:** Effect of *Nostoc sphaeroids* Kütz polysaccharide (NSKP) on bodyweight, kidney weight, and food intake in high‐fat mice at the end of 22 weeks

	Body weight (g)	Kidney weight (g)	Kidney weight/Body weight (%)	Food intake (g/day/mouse)
Cont	29.79 ± 1.52	0.35 ± 0.04	1.20 ± 0.14	3.99 ± 0.22
HFD	30.06 ± 1.81	0.36 ± 0.04	1.19 ± 0.06	3.68 ± 0.30
HFD+0.4 g/kg NSKP	31.76 ± 1.78	0.34 ± 0.03	1.08 ± 0.09	3.53 ± 0.21
HFD+0.8 g/kg NSKP	32.79 ± 2.07	0.40 ± 0.07	1.24 ± 0.15	3.61 ± 0.15

Note

Values were mean ± SEM (*n* = 10).

### 3.2 Effects of NSKP on serum biochemical indices

As shown in Table 3, the serum TC, TG, and LDL‐C in the HFD group were significantly increased and HDL‐C was significantly decreased compared with the Cont group (*p* < .05). The serum TC, TG, and LDL‐C were significantly decreased after treatment of NSKP, while HDL‐C was significantly increased (*p* < .05). The GLU of NSKP groups had a decreased tendency. In addition, the BUN and SCR after the NSKP treatment were significantly decreased (*p* < .05). The results showed that the NSKP had a good lipid‐lowering function in high‐fat mice, and also effectively reduced BUN and SCR.

**TABLE 3:** Effects of *Nostoc sphaeroids* Kütz polysaccharide (NSKP) on serum biochemical indices in high‐fat mice

	TC (mmol/L)	TG (mmol/L)	HDL‐C (mmol/L)	LDL‐C (mmol/L)	BUN (mmol/L)	SCR (mmol/L)	GLU (μmol/L)
Cont	14.66 ± 1.34^c^	2.6 ± 0.69^bc^	0.88 ± 0.18^a^	5.40 ± 0.95^c^	10.92 ± 1.30^b^	35.50 ± 4.25^c^	10.52 ± 1.49
HFD	28.10 ± 4.05^a^	9.61 ± 1.74^a^	0.55 ± 0.11^b^	28.27 ± 5.36^a^	21.87 ± 3.12^a^	53.3 ± 2.51^a^	9.39 ± 1.56
HFD+0.4 g/kg NSKP	21.29 ± 3.37^b^	5.33 ± 1.47^b^	0.86 ± 0.20^a^	12.57 ± 2.35^b^	8.69 ± 0.95^c^	42.88 ± 2.26^b^	8.25 ± 0.84
HFD+0.8 g/kg NSKP	21.51 ± 3.72^b^	3.84 ± 0.88^b^	0.72 ± 0.15^a^	13.45 ± 2.61^b^	8.71 ± 1.47^c^	41.75 ± 3.38^b^	8.31 ± 1.47

Note

Bars marked with different letters represent statistically significant difference (*p* < .05), whereas bars labeled with the same letter indicate no statistically significant difference between the groups (*p* > .05). Values represent mean ± SEM; *n* = 10 in each group.

Abbreviations: BUN, blood urea nitrogen; GLU, glucose; HDL‐C, high‐density lipoprotein‐ cholesterol; LDL‐C, low‐density lipoprotein‐cholesterol; NSKP, *Nostoc sphaeroids* Kütz polysaccharide; SCR, serum creatinine; TC, total cholesterol; TG, triglyceride.

### 3.3 H&E staining for renal cortex

**FIGURE 3:**
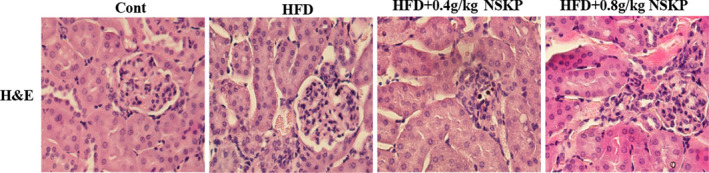
Effects of *Nostoc sphaeroids* Kütz polysaccharide (NSKP) on renal histopathology in high‐fat mice. Represents the H&E staining; original magnification: 400×

Renal tissue sections were stained with H&E. As shown in the H&E staining (Figure 3), the HFD group had some injury such as renal interstitium enlarged with increased cells, vacuolization of renal tubular epithelia, glomerular hypertrophy with the basal membrane thickened. After the NSKP treatment, the renal interstitium was significantly reduced, tubular cavitation was also reduced, and the glomerular basement membrane was narrowed. The results showed that NSKP could improve the structural abnormalities of renal cortex in high‐fat mice.

### 3.4 Effects of NSKP on F4/80, IL‐1β, and TNF‐α in renal inflammatory response

**FIGURE 4:**
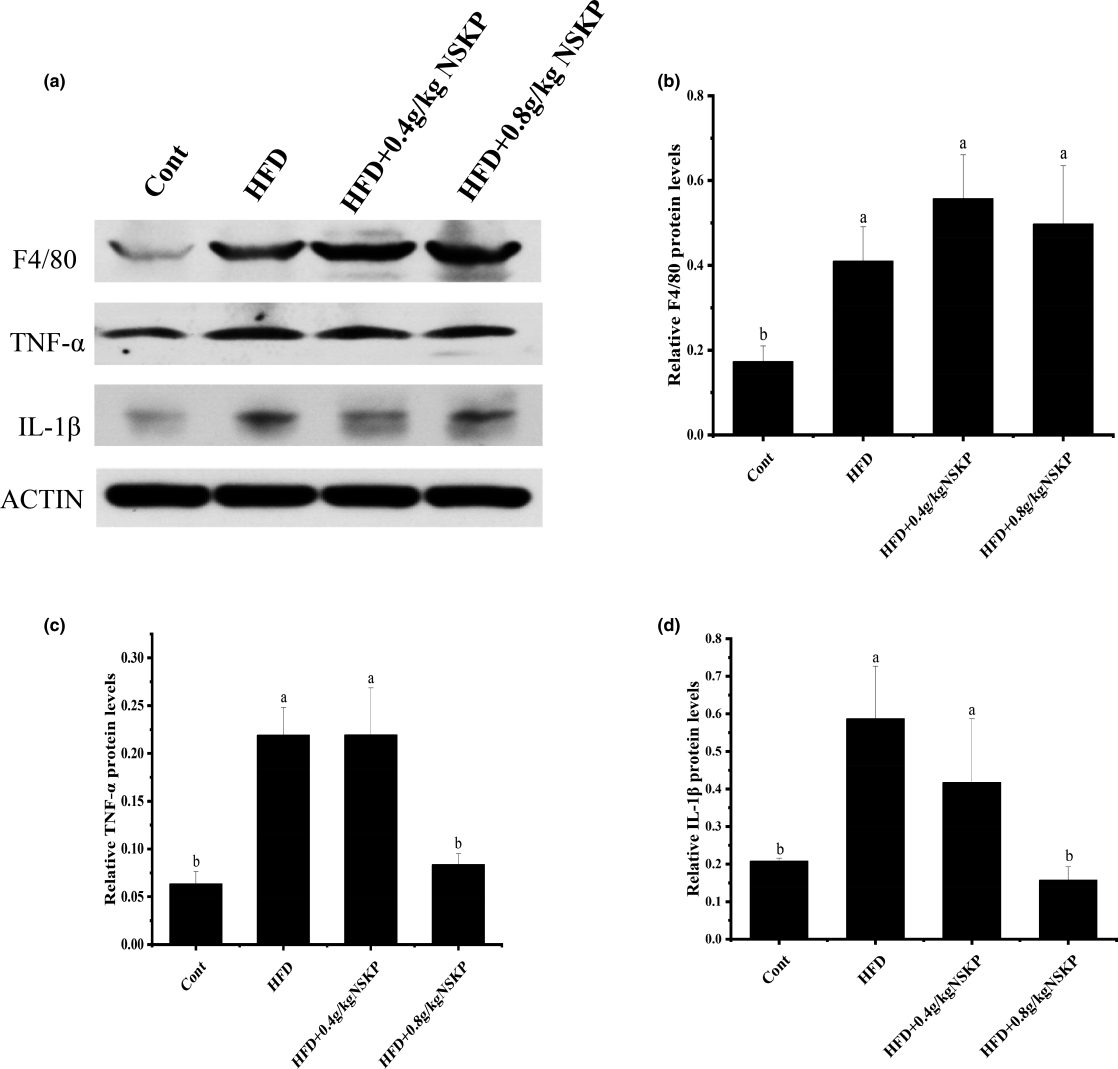
Effects of *Nostoc sphaeroids* Kütz polysaccharide (NSKP) on renal inflammatory reaction in high‐fat mice. (A) The blot of F4/80, TNF‐α and IL‐1β by Western blotting; (B‐D) protein expression of F4/80, TNF‐α, and IL‐1β. Bars marked with different letters represent statistically significant difference (*p* < .05), whereas bars labeled with the same letter indicate no statistically significant difference between the groups (*p* > .05). Values represent mean ± SEM; *n* = 8 in each group (F4/80 also called EMR 1: EGF‐like module‐containing mucin‐like hormone receptor‐like 1, TNF‐α: tumor necrosis factor‐alpha, IL‐1β: interleukin‐1 beta)

As shown in Figure 4a‐d, the macrophage marker F4/80 in the HFD group was significantly higher than in the Cont group (*p* < .05), but NSKP had no significant effect on renal macrophages in high‐fat mice. However, NSKP decreased the levels of inflammatory factors IL‐1β and TNF‐α in kidney of mice (*p* < .05). The results showed that NSKP could not reduce the macrophage infiltration caused by long‐term HFD, but could downregulate the expression of proinflammatory factors.

### 3.5 Effect of NSKP on expression of TGF‐β1 in renal cortex

**FIGURE 5:**
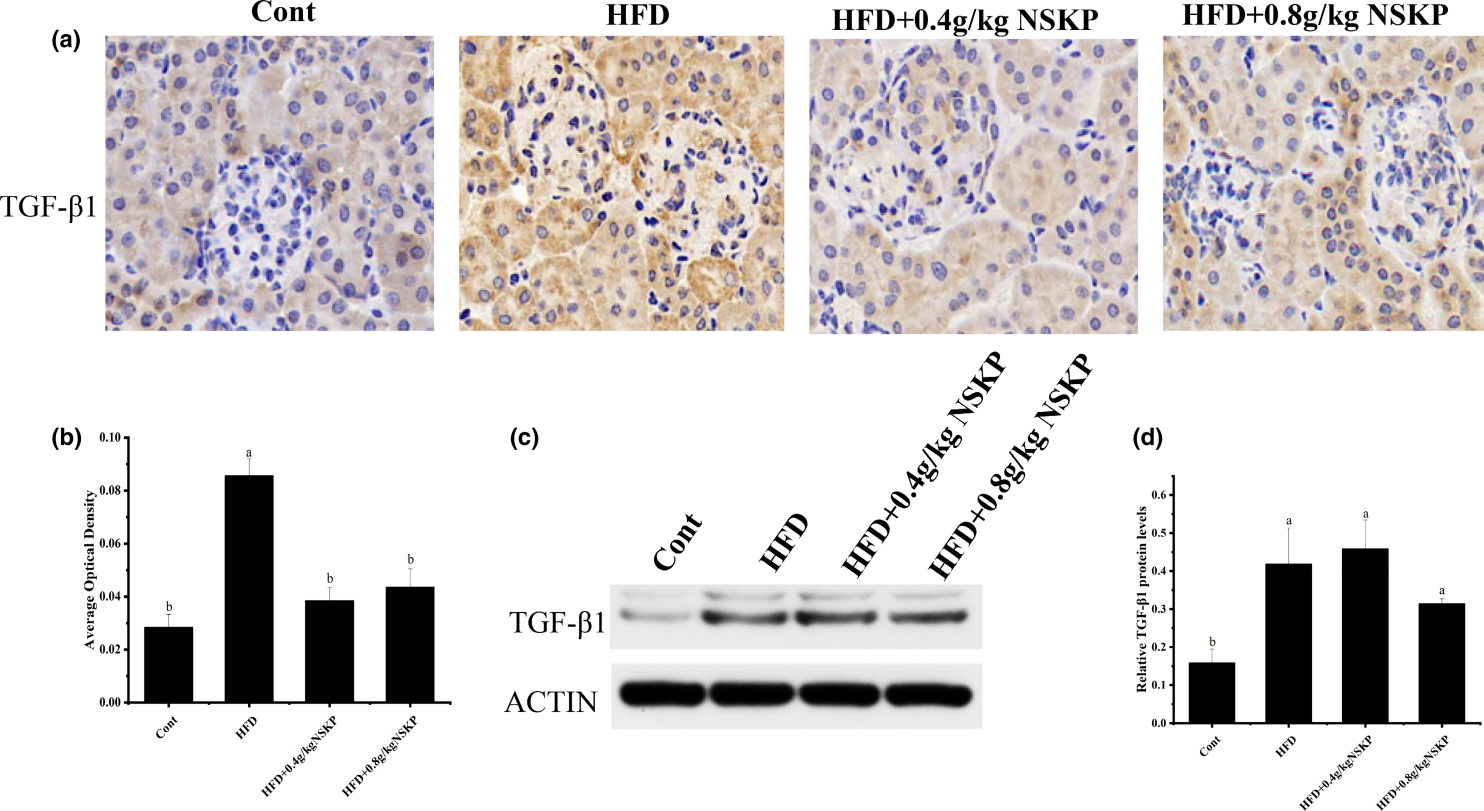
Effect of *Nostoc sphaeroids* Kütz polysaccharide (NSKP) on expression of TGF‐β1 in renal cortex. (A): Immunohistochemical staining, original magnification: 400×; (B) average optical density of TGF‐β1; (C) The blot of TGF‐β1 by Western blotting; (D) protein expression of TGF‐β1. Bars marked with different letters represent statistically significant difference (*p* < .05), whereas bars labeled with the same letter indicate no statistically significant difference between the groups (*p* > .05). Values represent mean ± SEM; *n* = 8 in each group (TGF‐β1: transforming growth factor‐beta 1)

As shown in Figure 5a,b, the yellow‐brown area and area optical density of TGF‐β1 in the HFD group were higher than in the Cont group, but the protein expression of renal TGF‐β1 in NSPK groups was significantly decreased (*p* <.05). The protein expression of TGF‐β1 in renal cortex was detected by Western blotting analysis technique and semi‐quantitative method. The TGF‐β1 protein expression of NSKP groups was not observed (Figure 5c,d). These results suggested that NSKP improved the expression of TGF‐β1 in the renal cortex of high‐fat mice.

### 3.6 Effects of NSKP on α‐SMA and FN, major markers of renal fibrosis

**FIGURE 6:**
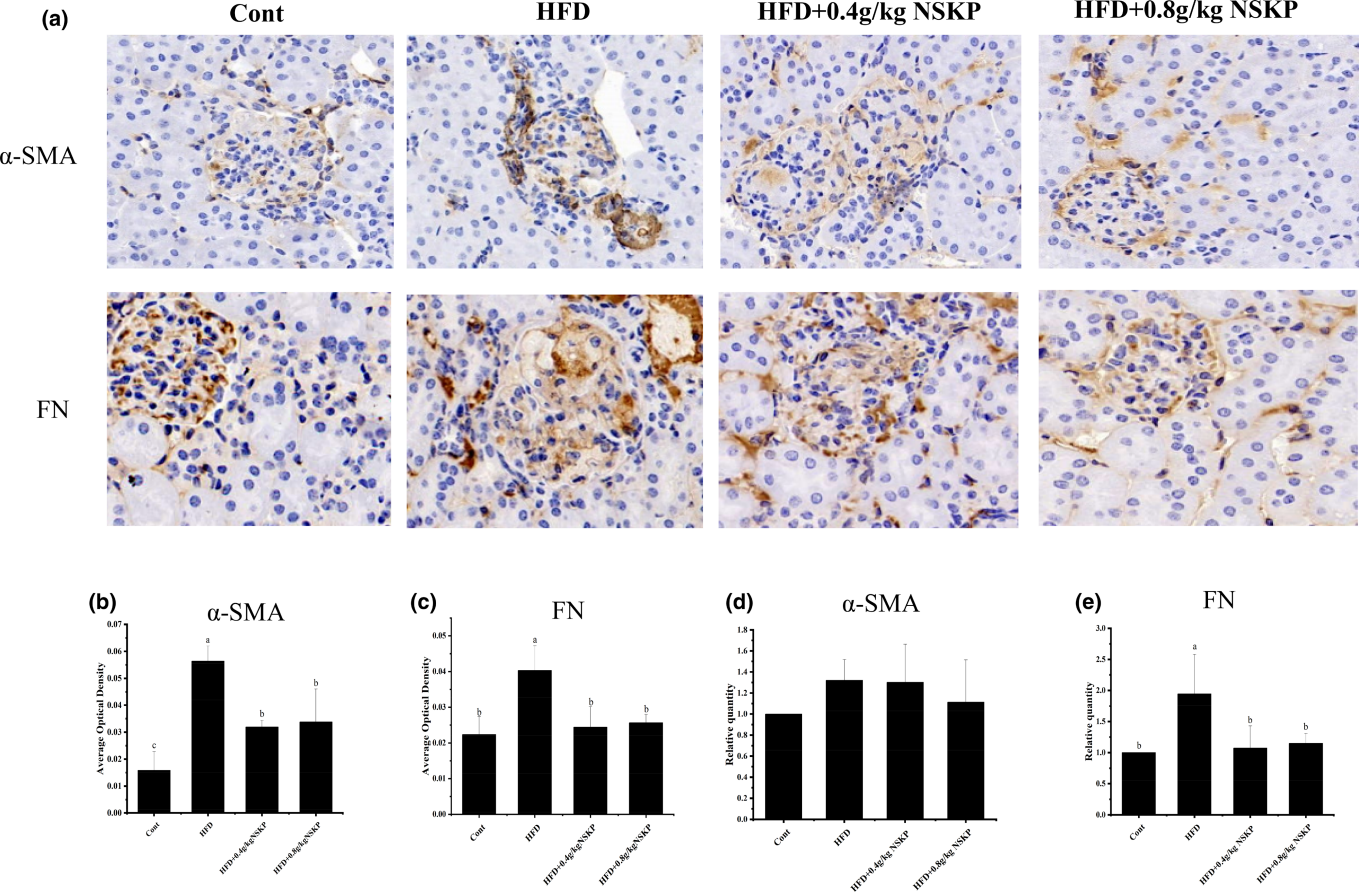
Effect of *Nostoc sphaeroids* Kütz polysaccharide (NSKP) on renal fibrosis markers α‐SMA and FN in high‐fat mice. (A): Immunohistochemical staining (α‐SMA, FN), original magnification: 400×; (B‐C) Quantitative analysis of the average optical density of immunohistochemical staining sections (α‐SMA, FN); (D‐E) the mRNA expression levels of α‐SMA and FN were detected by RT‐PCR. Relative quantity of α‐SMA and FN by RT‐PCR. Bars marked with different letters represent statistically significant difference (*p* < .05), whereas bars labeled with the same letter indicate no statistically significant difference between the groups (*p* > .05). Values represent mean ± SEM; *n* = 8 in each group (α‐SMA: α‐smooth muscle actin, FN: fibronectin)

On immune histochemical analysis, α‐SMA and FN from myofibroblasts were present in the renal glomerulus and tubule interstitium (Figure 6). As shown in Figure 6a‐c, α‐SMA and FN of the HFD group were overexpressed in the renal cortex compared with the Cont group. On the contrary, the expression of α‐SMA in renal cortex of mice treated with 0.4 g/kg BW NSKP was significantly decreased and also significantly decreased in mice treated with HFD+0.8 g/kg BW NSKP (*p* < .05). The expression of FN in the renal cortex of NSKP groups was significantly decreased (*p* < .05).

The mRNA expression levels of α‐SMA and FN in the renal cortex were detected by Real‐time polymerase chain reaction (RT‐PCR). As can be seen from Figure 6d‐e, the mRNA expression of α‐SMA and FN in the Cont group was significantly increased compared with the HFD group (*p* < .05). NSKP groups showed no significant difference in the mRNA expression of α‐SMA in the renal cortex of high‐fat mice. On the contrary, the mRNA expression of FN in the renal cortex of mice was significantly decreased by giving NSKP (*p* < .05). These results suggested that NSKP alleviated renal fibrosis in long‐term high‐fat mice through reducing the protein expression of α‐SMA and the deposition of FN in glomeruli and tubule interstitium.

### 3.7 Effects of NSKP on collagen fibers such as collagen I and collagen IV in renal cortex of high‐fat mice

**FIGURE 7:**
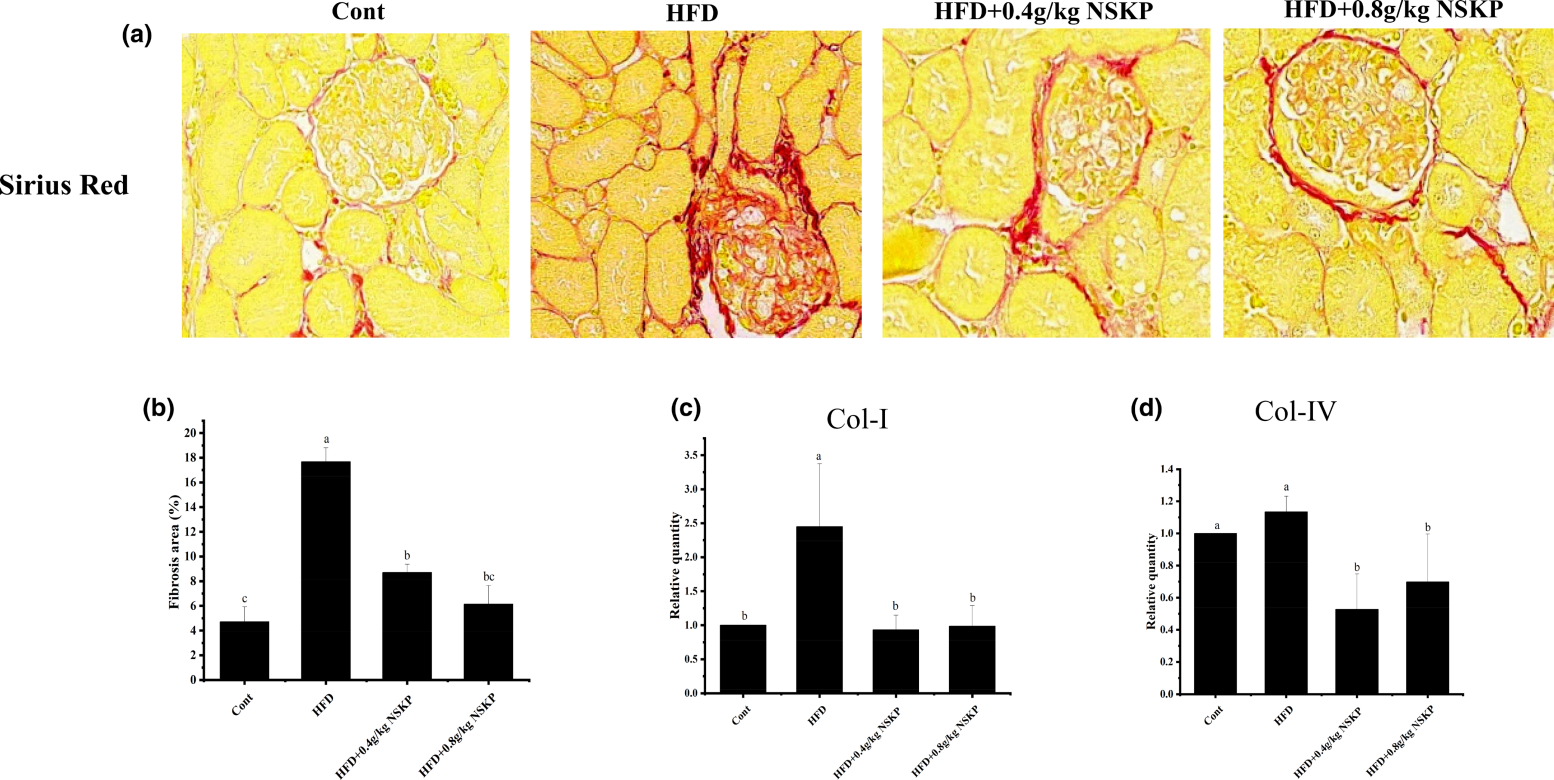
Effect of *Nostoc sphaeroids* Kütz polysaccharide (NSKP) on collagen fibers in high‐fat mice. (A) Sirius red staining of the renal cortex; red represents collagen fibers, and original magnification: 400×; (B) renal fibrosis area (%); (C‐D) the mRNA expression levels of Col‐I and Col‐IV were detected by RT‐PCR. Bars marked with different letters represent statistically significant difference (*p* < .05), whereas bars labeled with the same letter indicate no statistically significant difference between the groups (*p* > .05). Values represent mean ± SEM; *n* = 8 in each group (Col‐I: collagen I, Col‐IV: collagen IV)

Based on the correlation between collagen fibers in ECM and renal fibrosis, we performed Sirius Red staining on collagen fibers and analyzed the mRNA expression of Col‐I and Col‐IV. As shown in Figure 7a,b, the collagen fiber in the HFD group was significantly red and the collagen fiber deposition was serious compared with the Cont group. The collagen fibers in NSKP groups showed less red and less fibrosis area than in the HFD group, and the differences were significant (*p* < .05). As shown in Figure 7c, the mRNA expression of Col‐I in the HFD group was increased with a significant difference compared with the Cont group (*p* < .05). Conversely, the mRNA expression of that in the NSKP groups was significantly decreased compared with the HFD group (*p* < .05). As shown in Figure 7d, the mRNA expression of Col‐IV in the renal cortex of the HFD group tended to increase, but not significantly, compared with the Cont group. However, NSKP significantly decreased the mRNA expression of Col‐IV in the renal cortex of mice (*p* < .05). Therefore, NSKP reduced the collagen fiber deposition in the renal cortex of high‐fat mice, downregulated the mRNA expression of Col‐I and Col‐IV, and reduced the degree of renal fibrosis.

## 4 DISCUSSION AND CONCLUSIONS

A large number of studies have shown that dyslipidemia is caused by long‐term HFD, which can not only cause hyperlipidemia but also bring in complications caused by lipid metabolic disorders such as nonalcoholic fatty liver, atherosclerosis, insulin resistance, and renal injury. Epidemiology has consistently suggested that dyslipidemia caused by HFD is an important risk factor for CKD (Attman et al., 1999). Mice or rats fed a continuous high‐fat or high‐fat and sugar diet develop obesity and renal pathological changes, characterized by glomerulosclerosis, proteinuria, and interstitial fibrosis (Feng et al., 2017; Morrison et al., 2017; Reena & Kaur, 2016; Wang et al., 2017). The important mechanism lies in the increase in inflammatory factors caused by long‐term lipid metabolic disorders, which indicated that chronic inflammation is an important and potential factor to these diseases.

The complex polysaccharides derived from algae have a mitigating effect on the lesions caused by an HFD. For example, Maha et al. extracted polysaccharides from green algae that improved liver and kidney damage of mice caused by HFD. Chou et al. observed that inonotus obliquus polysaccharide (LIOP) improved renal fibrosis in diabetic nephropathy mice caused by glucose and lipid metabolism, and the mitigation mechanisms studied were all related to inhibition of inflammatory factors or activation of inflammatory signals (Chou et al., 2016). *Nostoc sphaeroids* Kütz is a kind of low‐grade unicellular cyanobacterium of cyanophyta and candidae. It has thousands of years for eating in China, and it is a rare natural pollution‐free green food. *Nostoc sphaeroids* Kütz has a good lipid‐lowering effect, which can directly reduce the absorption of cholesterol in the intestine and inhibit atherosclerotic lesions without increasing the burden of liver metabolism (Ku et al., 2015). Previous studies in our laboratory have proved that *Nostoc sphaeroids* Kütz can improve liver injury and intestinal microorganisms. These improvements mechanisms are related to the inhibition of the expression of inflammatory factors (Wei et al., 2019, 2020). The polysaccharide content in *Nostoc sphaeroids* Kütz is as high as 38%. The purity of NSKP used in our experiment reached 96.95%.

In our experiment, the serum TC, TG, and LDL‐C of HFD‐fed mice were significantly higher than those on normal standard diet, and HDL‐C was significantly lower, indicating that high fat diet caused the lipid metabolism disorder of ApoE^−/−^ mice. NSKP significantly reduced serum TC, TG, and LDL‐C in high‐fat mice. In addition, after long‐term treatment with NSKP, the SCR of high‐fat mice was also reduced, which directly indicated that NSKP had a lipid‐lowering effect on high‐fat mice, and also improved kidney injury caused by lipid metabolism disorder.

Excessive high‐fat food can lead to lipid metabolism disorder, activate the inflammation in the body, and cause inflammatory diseases in various organs (Murakoshi et al., 2021). This inflammation can cause the increase in inflammatory factors and attract the invasion of inflammatory cells such as lymphocytes and macrophages (Desai et al., 2017; Liu et al., 2014). Inflammatory cells are usually highly active in tissues and induce the production of more chemokines and inflammatory factors (such as monocyte chemoattractant protein 1 (MCP‐1), CCL2, TNF‐α, IL‐1β, and IL‐6), and this inflammatory signal is the main effect of renal fibrosis (Grande et al., 2015; Wynn & Vannella, 2016). In this experiment, we detected the renal inflammatory factors in high‐fat mice, which had received NSKP for a long time, and found that NSKP decreased the expression of renal inflammatory factors in mice.

TGF‐β1 is an important fibrogenic growth factor, which plays a key role in the process of fibrosis after lung injury. It is also known as the strongest hepatic fibrosis promoter, and it is also a key signal molecule of renal fibrosis (Willis & Borok, 2007; Zeisberg et al., 2008). This kind of cytokine mainly mediates the downstream Smad signal pathway, stimulates the activation of fibrosis mechanisms such as EMT and EndMT in renal intrinsic cells, and participates in renal fibrosis (Duffield, 2014; Zeisberg et al., 2008). The key target of nopiletine in the treatment of kidney disease was that it increased serum adiponectin level, upregulated the expression of adiponectin receptor in kidney tissue, suppressed the expression of TGF‐β1, and alleviated renal fibrosis (Bunbupha et al., 2020). The results of our experiment showed that NSKP downregulated the expression of renal inflammatory factors in high‐fat mice and slowed down the development of renal inflammation. And the expression of TGF‐β1, the key regulator of renal fibrosis in high fat‐fed mice, after NSKP treatment was significantly decreased, which improved renal fibrosis.

It is worth noting that this study found that NSKP had no effect on the mRNA expression of α‐SMA of myofibroblasts, but decreased protein expression, indicating that NSKP could improve renal fibrosis. Myofibroblasts are mainly derived from the transformation of intrinsic cells in kidney and promote the proliferation and activation of fibroblasts in glomeruli and renal interstitium. They are the main cells in renal fibrosis and have the function of synthesizing α‐SMA and secreting ECM (Liu, 2011). The invasion of macrophages and the massive activation of inflammatory response lead to excessive proliferation of myofibroblasts and stimulate myofibroblasts to overexpress α‐SMA and secrete ECM, such as FN, Col‐I, and Col‐IV (Parisi et al., 2018). The binding of TGF‐β1 to the receptor leads to the phosphorylation of Smad signal molecules, and the binding to the receptor results in the transfer of the whole complex to the nucleus to increase the gene expression of ECM (Meng et al., 2016). The existence of TGF‐ β1 also seriously affects the degradation mechanism of ECM, resulting in excessive deposition of ECM in glomeruli or renal interstitium, glomerular hypertrophy, and renal interstitial enlargement, injury of glomeruli and other tissue structures, and eventually renal fibrosis (Yu & Stamenkovic, 2000). Histopathological staining results showed that the renal tissue structure of HFD‐fed mice was abnormal, collagen fibers were seriously deposited in renal interstitium and glomeruli, and the degree of fibrosis was serious. However, the treatment of NSKP downregulated the mRNA expression of FN, Col‐I, and Col‐IV in high‐fat mice, slowed down the deposition of FN, Col‐I, and Col‐IV in the renal cortex of HFD mice, and improved renal fibrosis. Our study is only a preliminary exploration of the effect of NSKP on high‐lipid‐induced renal fibrosis. The specific mechanism of how NSKP works to protect the kidney from injury is still unclear.We only studied the effect of NSKP on renal inflammation in high‐fat mice. So, we still need to futher explore and study.

The NSKP reduced the blood lipid of long‐term high fat‐fed mice, downregulated the inflammatory response of kidney, inhibited the expression of collagen fiber, and improved the renal fibrosis caused by long‐term lipid metabolism disorder.

## CONFLICT OF INTEREST

No potential conflict of interest was reported by the author(s).

## AUTHOR CONTRIBUTIONS

**Litao Yang:** Investigation (equal); Methodology (equal); Validation (equal); Writing – original draft (equal). **Yinlu Liu:** Supervision (equal). **Cuicui Bi:** Supervision (equal). **Bo Zhang:** Supervision (equal).

## Data Availability

The data that support the findings of this study are openly available at [https://doi.org/10.1002/fsn3,2703].
